# Evaluación comparativa de las características de porosidad entre el cemento Portland, mta y Biodentine con microscopio electrónico de barrido

**DOI:** 10.21142/2523-2754-0901-2021-043

**Published:** 2021-03-11

**Authors:** Luis Manuel Bravo Eslava, César Antonio Gallardo Gutiérrez

**Affiliations:** 1 División de Carielogía y Endodoncia de la Carrera de Odontología, Universidad Científica del Sur. Lima, Perú. luisinho_447@hotmail.com, cgallardo03@yahoo.es Universidad Científica del Sur División de Carielogía y Endodoncia Carrera de Odontología Universidad Científica del Sur Lima Peru luisinho_447@hotmail.com cgallardo03@yahoo.es

**Keywords:** agregado de trióxido mineral, Biodentine, cemento Portland, microscopio electrónico de barrido, Mineral Trioxide Aggregate, Biodentine, Portland cement, Scanning electron microscope

## Abstract

**Objetivo:**

El propósito del presente estudio fue evaluar comparativamente las características de porosidad entre el cemento Portland, MTA Angelus® y Biodentine Septodont®, observados con un microscopio electrónico de barrido.

**Materiales y métodos:**

Se prepararon los cementos según las indicaciones del fabricante y se empaquetaron en tubos cilíndricos de polietileno con un diámetro interno de 10 mm y una altura de 5 mm. Se analizó la porosidad de las muestras mediante el microscopio electrónico de barrido. El análisis estadístico se realizó utilizando la prueba Kruskal-Wallis. El nivel de significancia se estableció en 0,05

**Resultados:**

: Se observó la descripción de la media de los valores del diámetro de los poros, y el tamaño mayor correspondió al cemento Portland (11,07). Existen diferencias significativas entre las medias del diámetro de los poros con un p = 0,05. Se identificó que el MTA Angelus® tiene la mayor cantidad de poros, le sigue el Biodentine Septodont® y, por último, el Portland. Se comparó la cantidad de poros entre los tres cementos y no se encontraron diferencias significativas, con un p = 0,09.

**Conclusión:**

Los análisis realizados en los cementos endodónticos dieron como resultado que el cemento Portland tiene mayor diámetro de poro a diferencia de los otros dos, lo cual implica que tanto el Biodentine Septodont® como el MTA Angelus® tienen mejores propiedades de resistencia y permeabilidad para evitar la microfiltración, y por tanto son mejores para la solución de casos clínicos.

## Abstract (en)

**Objective:**

The purpose of this study was to compare the porosity characteristics of Portland cement, mineral trioxide aggregate (MTA) Angelus® and Biodentine Septodont® by scanning electron microscopy.

**Materials and Methods:**

Cements were prepared according to the manufacturer's instructions and packed in cylindrical polyethylene tubes with an internal diameter of 10 mm and a height of 5 mm. The porosity of the samples was analyzed using scanning electron microscopy. Statistical analyses were performed using the Kruskall Wallis test. The level of significance was established at 0.05.

**Results:**

The largest size mean diameter valus was found with Portland cement (11.07). There were significant differences between the mean pore diameters (p = 0.05). MTA Angelus® had the largest number of pores, followed by Biodentine Septodont®, and finally, Portland. There were no significant differences in the pores of the three cements (p = 0.09).

**Conclusion:**

The results of this comparative analysis of endodontic cements showed that Portland cement has a larger pore diameter than MTA Angelus® and Biodentine Septodont®, demonstrating that these latter two cements present better resistance and permeability properties, and thereby prevent microleakage.

## INTRODUCCIÓN

Los materiales odontológicos han mejorado a través de los años gracias al avance de la tecnología, lo que les ha proporcionado excelentes propiedades físicas, químicas y biológicas ^1^. El material de reparación endodóntico debe tener radiopacidad, ser biocompatible, antibacteriano, poseer estabilidad dimensional, de fácil manipulación, osteoinductor y de sellado hermético ^2^. El cemento Portland se compone de 4 óxidos principales: cal (CaO), sílice (SiO2), alúmina (Al2O3) y óxido férrico (Fe2O3). La cal se obtiene por disgregación de la piedra caliza (CaCO3) y los otros componentes se producen a partir de esquisto (arsénico y plomo), por lo que no es aprobado para uso clínico ^3^.

Los radiopacificadores alternativos (sulfato de bario, óxido de zirconio, yodoformo, polvo de oro, dióxido de titanio, óxido de plomo, subnitrato de calcio, tungsteno de calcio, carbonato de bismuto y subnitrato de bismuto) se propusieron para superar las desventajas del bismuto como la genotoxicidad, la cual interfiere negativamente en la porosidad y la resistencia a la compresión ^4^.

El mineral trióxido agregado (MTA) es un material bioactivo hecho con silicato de calcio. Está compuesto en un 75% de cemento Portland, un 20% de óxido de bismuto, un 5% de yeso y cantidades mínimas de sulfato de sodio (Na2SO4), sulfato de potasio(K2SO4), óxido de silicio (SiO2), óxido de magnesio (MgO) y óxido de calcio (CaO). Su emplea clínicamente para la obturación retrógrada, la apicoformación, la apexificación, el recubrimiento pulpar, la revascularización la y reparación de perforaciones a nivel de la porción radicular del diente, pero tiene como desventajas el extenso tiempo de fraguado, la consistencia de la mezcla y la decoloración de los dientes ^5^^-^^10^.

Biodentine es un nuevo material biocompatible, bioactivo, fabricado con alita (Ca3SiO5) de alta pureza, compuesto por una parte sólida que contiene silicato tricálcico (3CaO SiO2), carbonato de calcio (CaCO3) y óxido de zirconio (ZrO2), y una parte líquida que contiene cloruro de calcio (CaCl2) y un agente reductor de agua. Puede estimular la regeneración de la dentina mediante la inducción de odontoblastos y la diferenciación de las células progenitoras de la pulpa. Sus características de fraguado y comportamiento mecánico lo hacen apropiado como un sustituto de la dentina ^11^^,^^12^.

La porosidad es una medida de los espacios vacíos en una sustancia. Puede aumentar la permeabilidad del material fraguado y tener un impacto en muchos otros factores, como la absorción, la permeabilidad, la resistencia y la densidad. Puede actuar como un portal de entrada para microorganismos por microfiltración y está directamente relacionada con el comportamiento de filtración del material de relleno del conducto. La porosimetría es la medición del volumen, el tamaño, la densidad y la porosidad de distribución de un material ^13^.

Saghiri et al. (2017) encontraron que MTA mostró menor porosidad en comparación con Biodentine ^13^. Formosa et al. (2014) hallaron que el MTA plus mezclado con gel antilavado tiene una porosidad inicial más baja que MTA plus mezclado con agua ^14^. Guerrero et al. (2018) obtuvieron, por medio de la microtomografía computarizada, que Biodentine tiene una porosidad más baja que ProRoot MTA ^1^. Chang (2018) demostró que Biodentine y MTA Angelus tienen menor diámetro de poros ^15^.

De esta manera, se ha informado que las investigaciones de las pruebas mecánicas sencillas permiten una correlación de las propiedades mecánicas con el rendimiento clínico, y pueden aconsejar a los odontólogos que los cementos necesitan cuidados especiales o conllevan riesgos parti-culares durante la mezcla y colocación del material ^16^.

La evaluación de las propiedades físicas de Biodentine demostró que la adición de aditivos a los cementos con base en silicato tricálcico influye en las propiedades físicas de los materiales ^17^. Por lo tanto, el propósito del presente estudio es evaluar comparativamente las características de porosidad entre el cemento Portland, MTA y Biodentine, mediante un microscopio electrónico de barrido.

## MATERIALES Y MÉTODOS

Este estudio fue aprobado por el Comité Institucional de Ética de la Universidad Científica del Sur (CIEI-Científica), con el número de protocolo: 255-2019-POS8. Este estudio descriptivo transversal, fue realizado en el laboratorio de equipamiento especializado de la Facultad de Ciencias Biológicas UNMSM en el período de agosto del 2019. 

La muestra consistió en 5 cilindros de MTA, 5 de Biodentine y 5 de cemento Portland. Para determinar el tamaño de la muestra, se utilizó la fórmula de comparación de medias con el dato de la varianza de la prueba piloto, con un nivel de confianza del 95% y un margen de error del 5%, lo que dio como resultado 5 muestras por grupo. Se consideraron cajas de cemento con fecha de expiración dentro del tiempo permisible y se excluyeron aquellos empaques adulterados y los cementos previamente utilizados.

Se empleó el cemento Portland Cemento Sol® (Perú), MTA Angelus® (Brasil) y Biodentine Septodont® (Francia). Los cementos fueron mezclados de acuerdo con las instrucciones del fabricante. Así, se mezcló en un vaso pírex 1 g de cemento Portland con 1 ml de agua, utilizando una espátula metálica. Le mezcla resultante tuvo una consistencia pastosa y fue empaquetada con un portaamalgama en un tubo cilíndrico de polietileno con un diámetro interno de 10 mm y una altura de 5 mm, para dejarlo fraguar por 10 horas.

En el caso del MTA, se mezcló 1 g de este material con una gota de agua destilada en una platina de vidrio utilizando una espátula metálica durante 30 segundos. El resultado mostró una consistencia arenosa y se empaquetó con un portaamalgama en un tubo cilíndrico de polietileno con un diámetro interno de 10 mm y una altura de 5 mm, para dejarlo fraguar por 15 minutos.

**Figura 1:**
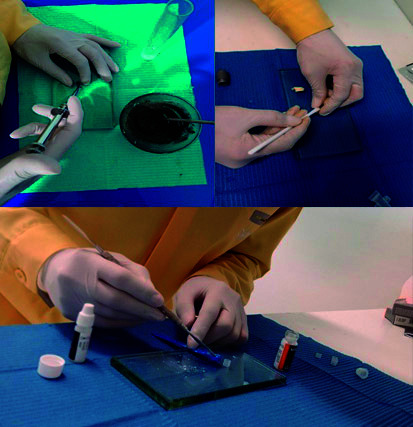
Preparación de los cementos.

Por su parte, se colocó dentro de la cápsula de Biodentine 5 gotas cloruro de calcio y se mezcló con un amalgamador durante 30 segundos. El resultado mostró una consistencia espesa y se empaqueto con un portaamalgama en un tubo cilíndrico de polietileno con un diámetro interno de 10 mm y una altura de 5 mm, para dejarlo fraguar por 12 minutos (Figura 1).

**Figura 2:**
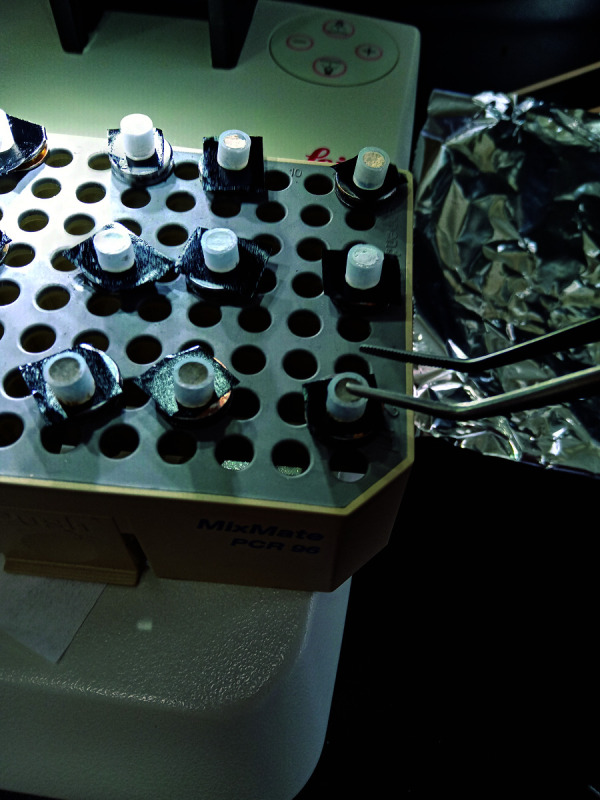
Fijación de las muestras en las plataformas metálicas.

**Figura 3:**
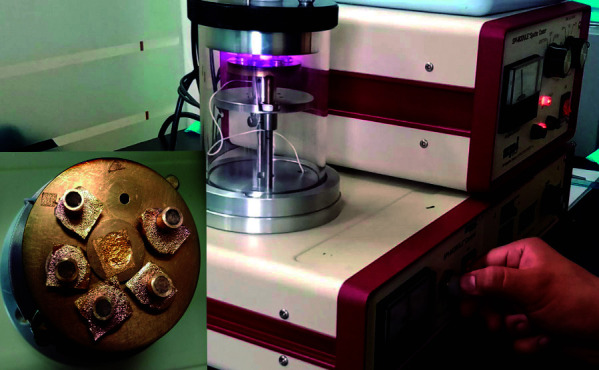
Metalizado de las muestras.

Luego, las muestras se fijaron en cinta de carbono en plataformas de metal denominadas *stub* (Figura 2). Se realizó el metalizado por *sputter*, mediante el bañado en oro (1 minuto y 30 segundos a 18 miliamperios) y se evaluó con un microscopio electrónico de barrido marca FEI, modelo Inspect S50 (República Checa) (Figura 3). 

**Figura 4:**
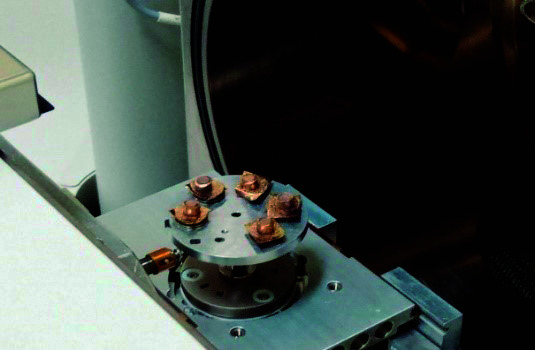
Evaluación de los cementos endodónticos en el microscopio electrónico de barrido.

Para enfocar con el MEB los cilindros de cada cemento, estos se dividieron en cuadrantes y se seleccionó un cuadrante por muestreo aleatorio simple de cada muestra para su análisis. Se tomaron y analizaron las microfotografías a 5000 aumentos (Figura 4).

El investigador se capacitó en el manejo del programa Image J para el cálculo del número y el diámetro de poros de cada cuadrante. Se aplicó la prueba estadística de coeficiente de correlación intraclase, y el coeficiente de correlación interobservador fue 0,98 y el de correlación intraobservador fue 1. 

El número de poros se calculó utilizando el programa Image J versión 1.52 (Rasband WS, ImageJ; Instituto Nacional de Salud de EE. UU., Bethesda, MD, EE. UU.). Cada figura fue invertida mediante este programa y el brillo se ajustó para seleccionar los orificios de cada figura, que fueron contabilizados por este para calcular el número total de poros. 

**Figura 5:**
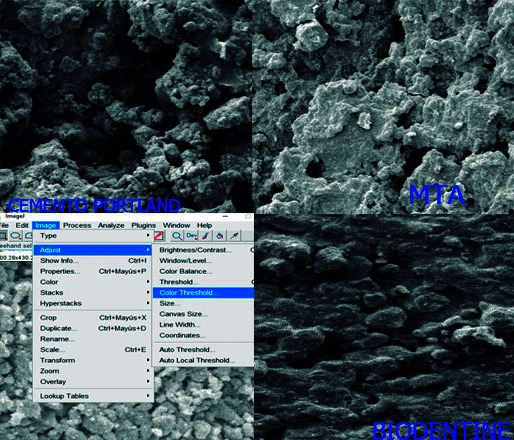
Microfotografías a 5000 X aumentos de cada muestra ingresadas y analizadas en el programa Image J.

El diámetro de poros se calculó utilizando el programa Image J y cada poro se midió con una regla virtual que arrojó una medida en µm (Figura 5).

### Análisis estadístico

Se realizó el análisis de Kruskal-Wallis, mediante el programa estadístico SPSS versión 25.0, del año 2017. Para ello se empleó la estadística descriptiva y se obtuvo media, desviación estándar, valor menor y valor mayor de la variable, así como número y diámetro de poros.

Se utilizó estadística inferencial mediante la prueba de Kruskal-Wallis con una significancia 0,05 para comparar el número y el diámetro de poros de los cementos Portland, MTA y Biodentine.

## RESULTADOS

En la Tabla 1 se puede observar la descripción de la media de los valores del diámetro de los poros, y se observa el mayor tamaño en el cemento Portland (11,07 µm), seguido por el Biodentine (9,21 µm) y, por último, el MTA (8,41 µm). En la Tabla 2 se puede ver que existen diferencias significativas entre las medias del diámetro de los poros con un p = 0,03. En la Tabla 3 se identifica que el MTA tiene la mayor cantidad de poros (2925,20 µm^2^), seguido por el Biodentine (2062,00 µm^2^) y el cemento Portland (1139,60 µm^2^). En la Tabla 4, al comparar la cantidad de poros entre los tres cementos, no se encuentra diferencias significativas con un p = 0,09.

**Tabla 1:** Descripción de los valores del diámetro en micras de los poros de los cementos MTA, Portland y Biodentine

Valores	Media	Desviación	Mínimo	Máximo
Biodentine	9,21	4,88	2,92	28,02
MTA	8,41	6,06	2,17	27,83
Portland	11,07	11,51	2,92	92,64

**Tabla 2:** Evaluación comparativa de las características de porosidad del diámetro de los poros de los cementos

Cementos	Media	P
Biodentine	9,21	0,03
MTA	8,41	
Portland	11,07	

Prueba Kruskal-Wallis

**Tabla 3:** Descripción de los valores del número de poros en micras cuadradas de los cementos Biodentine, Portland y MTA

Valores	Media	Desviación	Mínimo	Máximo
Biodentine	2062,00	1550,10	739,00	4593,00
MTA	2925,20	1670,43	1121,00	4794,00
Portland	1139,60	833,78	526,00	2586,00

**Tabla 4:** Evaluación comparativa de las características de porosidad del número de poros de los cementos Biodentine, MTA y Portland

Cementos	Media	P
Biodentine	2062,00	0,09
MTA	2925,20	
Portland	1139,60	

Prueba Kruskal-Wallis

## DISCUSIÓN

En el presente estudio se evaluó materiales de tipo biocerámicos, que han demostrado mejores propiedades tanto biológicas como mecánicas al ser utilizados en contacto directo con tejidos periapicales.

Para este estudio, se utilizó el programa Image J que sirve para muchos tipos de mediciones. Esta herramienta se utiliza en el área de histología para las mediciones de células.

Los resultados obtenidos muestran, en la descripción de la media de los valores del diámetro de los poros, un mayor tamaño en el cemento Portland (11,07 µm), seguido por Biodentine (9,21 µm) y MTA (8,41 µm). En las medias del diámetro de los poros, sí existe una diferencia estadísticamente significativa con un p = 0,03. En cuanto a la descripción de la media de los valores del número de los poros, el MTA tiene la mayor cantidad de poros (2925,20 µm^2^), seguido por Biodentine (2062,00 µm^2^) y Portland (1139,60 µm^2^). Al comparar las medias del número de poros entre los tres cementos, no existe diferencia estadísticamente significativa (p = 0,09).

En el presente estudio, los resultados son similares a los obtenidos por Guerrero et al. (2018), pues ambos estudios dan como resultado un mayor número de poros en el MTA ^1^.

La porosidad de MTA es mayor que la de Biodentine. Estos valores difieren de los encontrados por Saghiri et al. (2017), quienes hallaron que el MTA mostró una menor porosidad de la superficie en comparación con Biodentine. Posiblemente, esta diferencia se debe a que en su metodología sumergieron los tubos en agua destilada y fluido sintético de tejido ^13^.

En el estudio también se obtuvo que el cemento Portland tiene un mayor diámetro de poros, lo que coincide con los resultados obtenidos por Antonijevic et al. (2014). No obstante, sus resultados incluyeron diferentes cantidades de radiopacificadores en el cemento Portland ^4^.

La investigación efectuada por Chang (2018) encontró que el cemento Portland tiene mayor diámetro de poros que Biodentine y MTA, lo que coincide con nuestros resultados ^15^.

Las características de porosidad se evaluaron de acuerdo con el número y el diámetro de los poros. El cemento Portland presentó los poros con mayor diámetro, mientras que el MTA y Biodentine presentaron mayor cantidad de poros, pero de menor calibre.

Los poros de menor diámetro proporcionan a los cementos endodónticos una mejor resistencia mecánica y permeabilidad para evitar la microfiltración de bacterias y sus toxinas en la dentina ^4^.

Asimismo, en la investigación se empleó el microscopio electrónico de barrido para medir la porosidad en diferentes cementos endodónticos, aunque sería conveniente emplear otro tipo de equipo, por ejemplo, el porosímetro, que nos puede dar otro panorama sobre la porosidad.

La importancia de este estudio radica en dar a conocer las características de la porosidad de los cementos endodónticos, lo que puede orientar al clínico para una mejor selección del material, por su resistencia mecánica y permeabilidad, en el tratamiento de los diferentes casos clínicos, como perforaciones radiculares, recubrimientos pulpares, cirugías apicales y reabsorciones internas y externas.

## CONCLUSIONES

Se concluye que el cemento Portland, al presentar un mayor diámetro de poros, podría ser menos resistente a la compresión, a diferencia de los cementos Biodentine y MTA. En cuanto al número de poros, no se halló una diferencia significativa entre los cementos Portland, Biodentine y MTA.

## Fuente de financiamiento:

Autofinanciado
